# Long-Term Mortality Risk According to Cardiorespiratory Fitness in Patients Undergoing Coronary Artery Bypass Graft Surgery

**DOI:** 10.3390/jcm13030813

**Published:** 2024-01-31

**Authors:** John Duggan, Alex Peters, Jared Antevil, Charles Faselis, Immanuel Samuel, Peter Kokkinos, Gregory Trachiotis

**Affiliations:** 1 Department of Surgery, Walter Reed National Military Medical Center, Bethesda, MD 20814, USA; 2 Division of Cardiothoracic Surgery and Heart Center, Washington DC Veterans Affairs Medical Center, Washington, DC 20422, USA; 3 Cardiology Division, Washington DC Veterans Affairs Medical Center, Washington, DC 20422, USA; charles.faselis@va.gov (C.F.); peter.kokkinos@va.gov (P.K.); 4 War Related Illness and Injury Study, Washington DC Veterans Affairs Medical Center, Washington, DC 20422, USA; 5 Henry M. Jackson Foundation for the Advancement of Military Medicine, Bethesda, MD 20817, USA; 6 Department of Kinesiology and Health, Rutgers University, New Brunswick, NJ 08901, USA; 7 Department of Clinical Research and Leadership, George Washington University, Washington, DC 20037, USA; 8 Division of Cardiothoracic Surgery, The George Washington University Medical Center, Washington, DC 20422, USA

**Keywords:** cardiorespiratory fitness, coronary artery disease, coronary artery bypass grafting

## Abstract

The aim of this study was to evaluate the association between cardiorespiratory fitness (CRF) and long-term survival in United States (US) Veterans undergoing CABG. We identified 14,550 US Veterans who underwent CABG at least six months after completing a symptom-limited exercise treadmill test (ETT) with no evidence of cardiovascular disease. During a mean follow-up period of 10.0 ± 5.4 years, 6502 (43.0%) died. To assess the association between CRF and risk of mortality, we formed the following five fitness categories based on peak workload achieved (metabolic equivalents or METs) prior to CABG: Least-Fit (4.3 ± 1.0 METs (n = 4722)), Low-Fit (6.8 ± 0.9 METs (n = 3788)), Moderate-Fit (8.3 ± 1.1 METs (n = 2608)), Fit (10.2 ± 0.8 METs (n = 2613)), and High-Fit (13.0 ± 1.5 METs (n = 819)). Cox proportional hazard models were used to calculate risk across CRF categories. The models were adjusted for age, body mass index, race, cardiovascular disease, percutaneous coronary intervention prior to ETT, cardiovascular medications, and cardiovascular disease risk factors. *P*-values < 0.05 using two-sided tests were considered statistically significant. The association between cardiorespiratory fitness and mortality was inverse and graded. For every 1-MET increase in exercise capacity, the mortality risk was 11% lower (HR = 0.89; CI: 0.88–0.90; *p* < 0.001). When compared to the Least-Fit category (referent), mortality risk was 22% lower in Low-Fit individuals (HR = 0.78; CI: 0.73–0.82; *p* < 0.001), 31% lower in Moderate-Fit individuals (HR = 0.69; CI: 0.64–0.74; *p* < 0.001), 52% lower in Fit individuals (HR = 0.48; CI: 0.44–0.52; *p* < 0.001), and 66% lower in High-Fit individuals (HR = 0.34; CI: 0.29–0.40; *p* < 0.001). Cardiorespiratory fitness is inversely and independently associated with long-term mortality after CABG in Veterans referred for exercise testing.

## 1. Introduction

Coronary artery disease (CAD) is the leading cause of death in the United States (US) and represents a tremendous burden to the US healthcare system [1]. The quantity of years of life lost due to premature mortality from CAD is greater than the sum of that from lung cancer, colon cancer, breast cancer, and prostate cancer [2]. The incidence of CAD and death from CAD are expected to continue to rise in the coming decades [3]. For many patients, coronary artery bypass grafting (CABG) provides long-term survival benefits far in excess of what is achievable via medical management or percutaneous intervention [4,5]. Tremendous progress has been made in the medical and surgical management of CAD over the past several decades, leading to excellent short-term and long-term mortality after CABG. Risk factors associated with 30-day mortality after CABG have been well studied and accurately modeled in the Society of Thoracic Surgeons (STS) Risk Calculator; however, patient factors associated with long-term mortality have not been as well defined [6,7,8]. Though many comorbidities such as chronic kidney disease, diabetes mellitus, obesity, and smoking have been investigated as risk factors for late mortality after CABG, preoperative cardiorespiratory fitness (CRF) has not been adequately studied [9,10].

Cardiorespiratory fitness is widely recognized as a powerful predictor of all-cause mortality in patients with and without cardiovascular disease (CVD) [11,12]. CRF has also been noted to have an inverse and graded association with the development of many adverse health conditions, including CAD, CVD events including myocardial infarction, and heart failure [11,13,14,15,16,17]. A study using the STS database found a significant association between low fitness and 30-day mortality after CABG; however, the association between CRF and long-term outcomes after CABG has not been adequately studied [18]. To date, the only study to investigate the relationship between CRF and survival after CABG relied on patient reports of physical activity to estimate fitness levels and did not include direct objective measures of CRF such as treadmill exercise tolerance testing [19].

The demonstration of an inverse relationship between CRF and long-term mortality after CABG would provide clinicians with more evidence to promote exercise and physical fitness to their patients and to the public. CAD has immense impact on the health of the US population and on health expenditures, including revascularization. Illuminating the relationship between CRF and survival after CABG would provide additional prognostic information about the long-term risks and benefits of CABG, thereby assisting patients, surgeons, and other clinicians in shared decision-making regarding the optimal management of CAD. The aim of the present study is to investigate the association between preoperative CRF and long-term survival in United States Veterans undergoing CABG.

## 2. Materials and Methods

### 2.1. Study Population

**Figure 1:**
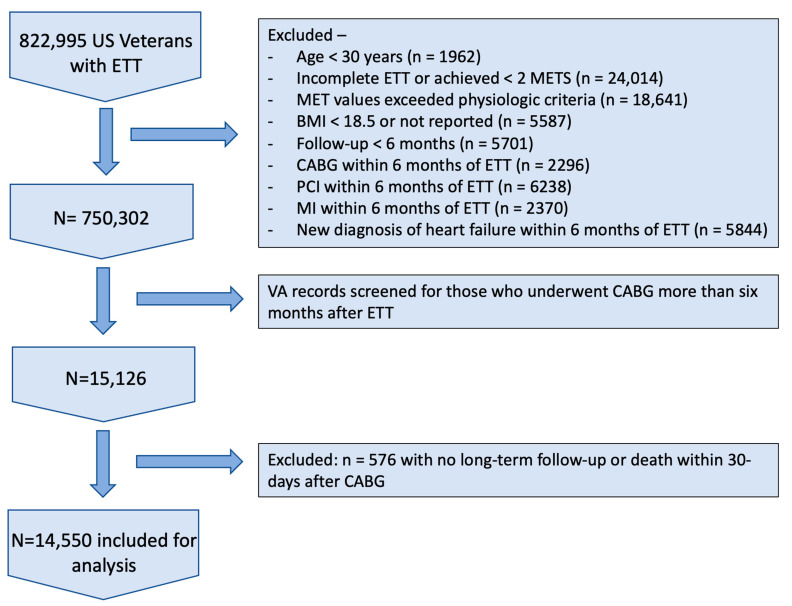
Flow chart of study population selection with exclusion criteria. US = United States; ETT = Exercise Tolerance Test; METS = Metabolic Equivalents; BMI = Body Mass Index; CABG = Coronary Artery Bypass Grafting; PCI = Percutaneous Coronary Intervention; MI = Myocardial Infarction; VA = Veterans Affairs.

The cohort was derived from the Exercise Testing and Health Outcomes Study (ETHOS) led by the Veterans Affairs Medical Center in Washington, DC (DCVAMC). The ETHOS study was initiated in April of 2016 and evolved as data mining tools became available and more powerful. The main aim of the study was to investigate associations between CRF and health outcomes. Accordingly, any clinical assessments and data we extracted before the study initiation date were considered retrospective, while data captured after the study initiation date were considered prospective. We identified 822,995 US Veterans who underwent an ETT performed within Veterans Affairs hospitals across the USA between 1 October 1999 and 3 September 2020 using the Bruce protocol. The reason for referral for the ETT was not known. The goals of ETT with the Bruce protocol are not to determine the presence or absence of cardiac ischemia but rather to quantify aerobic capacity. Exercise tolerance testing is terminated at the development of signs or symptoms of cardiac ischemia; any such test was considered incomplete and was not included in our study. Figure 1 illustrates how the study population was selected. We excluded 72,693 subjects who met the following criteria: (1) individuals < 30 years of age at the time of the ETT (n = 1962); (2) individuals who did not achieve a maximal effort (ETT was deemed incomplete as stated in the medical notes), achieved < 2.0 METs (n = 24,014), or achieved MET values that exceeded physiologic criteria (n = 18,641); (3) those with body mass index (BMI) < 18.5 g/m^2^ or missing BMI (n = 5587); and (4) those with a follow-up period < 6 months (n = 5701). To lower the likelihood of including individuals with overt heart disease that would limit exercise capacity and measured CRF, we also excluded those who met the following conditions within 6 months post-ETT: (1) underwent coronary artery bypass grafting (CABG; n = 2296), (2) underwent percutaneous coronary intervention (PCI; n = 6238), (3) experienced myocardial infarction (MI; n = 2370), or (4) received a diagnosis of chronic heart failure (CHF; n = 5884). After these exclusions, the final cohort consisted of 750,302 subjects (705,163 men and 45,139 women). Of those, 552,922 (73.7%) were white; 142,798 (19.0%) African-American; 35,197 (4.7%) Hispanic; 16,050 (2.1%) Native-American, Asian, or Hawaiian; and 3335 (0.4%) declined to report. This study was conducted under the supervision of the Washington DC Veterans Affairs Medical Center IRB, IRBNet ID 1584919-3, initially approved 12/2/2011.

Detailed information on relevant demographic, clinical, and medication information; risk factors; and comorbidities as defined by ICD9 and ICD10 coding, with at least 2 recordings at least 6 months apart, were obtained for all participants from the VA Computerized Patient Record System at the time of the ETT. The VA records have a high sensitivity for the incidence of chronic conditions [20,21]. Historical information included onset of previous myocardial infarction, cardiac procedures, heart failure, hypertension, diabetes mellitus, hypercholesterolemia, cancer (all), renal disease, stroke, smoking status (current and past), aspirin, and use of cardiac/antihypertensive medications. Data and analyses are presented in accordance with the Strengthening the Reporting of Observational Studies in Epidemiology (STROBE) reporting guidelines for cohort studies [22].

### 2.2. MET Extraction

We randomly selected 3000 samples of physician clinical notes on exercise capacity from the dataset and identified METs manually. This annotated dataset was further preprocessed and then used to train Natural Language Processing models. In the preprocessing phase, we removed special characters ($, &, etc.) and restricted the note to 30 characters before and after the words METs or MET. These words were then replaced with a special character to identify their location within the notes. Spacy software was then used to convert the resulting string into word tokens and then to a vector of numbers. Corresponding labels were created such that 1 meant that the corresponding token contained the MET value and 0 meant that it did not. We used a two-layer convolutional neural network using the Tensorflow software library to predict the probable location of METs in the notes. The model was trained over 100 epochs. Once METs were extracted, the MET data were randomly and manually checked for errors. The model accuracy on the test dataset was 97%.

### 2.3. CABG and Mortality

Participants were cross-referenced with the VA Informatics and Computing Infrastructure (VINCI) database for incidence of CABG using standard CPT codes. Patients who underwent CABG less than six months after ETT were excluded to further ensure that all ETTs were limited by exercise capacity and not by cardiac ischemia. We identified 15,126 patients who underwent CABG. A total of 576 (3.8%) did not have long-term follow-up data available and were excluded from the survival analysis. Patients who died in the perioperative period, defined as within 30 days of surgery, were excluded from the long-term mortality analysis. We identified 6502 patients who died in the follow-up period, representing 43.0% of the total cohort. Date of death was determined via the VINCI database, which is linked with Veterans Health Administration vital status files, the Social Security Administration, the Center for Medicare and Medicaid Services, and the National Cemetery Administration. Follow-up was completed through 30 September 2021 and is reported as mean +/− standard deviation, determined from the date of ETT to the date of death or last reported medical visit. 

### 2.4. CRF Categories

Peak MET levels were calculated for each participant using standardized American College of Sports Medicine equations based upon treadmill speed and grade [23]. To determine the age-specific CRF categories, we stratified the cohort into five age groups (30–49, 50–59, 60–69, 70–79, and 80–95 years) and established five CRF categories within each age group using methods described in our previous work [24]. We identified subjects with exercise performance less than 20%, 21–40%, 41–60%, 61–80%, and greater than 80% of predicted CRF within their respective age groups. The following five fitness quintiles were formed: Least-Fit (4.3 ± 1.0 METs (n = 4722)), Low-Fit (6.8 ± 0.9 METs (n = 3788)), Moderate-Fit (8.3 ± 1.1 METs (n = 2608)), Fit (10.2 ± 0.8 METs (n = 2613)), and High-Fit (13.0 ± 1.5 METs (n = 819)). 

Cox proportional hazard models were used to calculate the mortality risk for each CRF category. The models were adjusted for age, body mass index, race, cardiovascular disease including stroke and peripheral vascular disease, percutaneous transluminal coronary angioplasty prior to ETT, cardiovascular medications, time from ETT to CABG, and cardiovascular disease risk factors including hypertension, diabetes, chronic kidney disease, smoking, and dyslipidemia. Using the Least-Fit group as a reference, relative risk of mortality was assessed across the five CRF categories. Comparisons between categorical variables were evaluated using chi-squared tests, and continuous variables were assessed with one-way ANOVA tests. All statistical analyses were performed using SPSS software version 26 (IBM SPSS Statistics for Windows, Version 26; IBM Corp, Armonk, NY, USA).

## 3. Results

Clinical and demographic data at the time of ETT are presented in Table 1. Patients in the lower CRF quintiles tended to be older, have higher body weight and body mass index (BMI), and more frequently have chronic medical conditions such as diabetes mellitus, hypertension, or chronic kidney disease. The amount of time from ETT to CABG differed slightly among the CRF quintiles: Least-Fit = 4.7 ± 3.6 years, Low-Fit = 5.2 ± 3.5 years, Moderate-Fit = 5.4 ± 3.6 years, Fit = 5.4 ± 3.5 years, High-Fit = 5.9 ± 3.6 years. The mean follow-up time after CABG was 10.0 ± 5.4 years.

Patient factors that had a significant association with mortality risk after CABG are presented in Table 2. Notably, patient characteristics associated with improved survival included a history of PCI (HR 0.83, 95% confidence interval 0.75–0.93, *p* = 0.001) and statin use at the time of ETT (HR 0.93, 95% confidence interval 0.89–0.99, *p* = 0.012).

**Figure 2:**
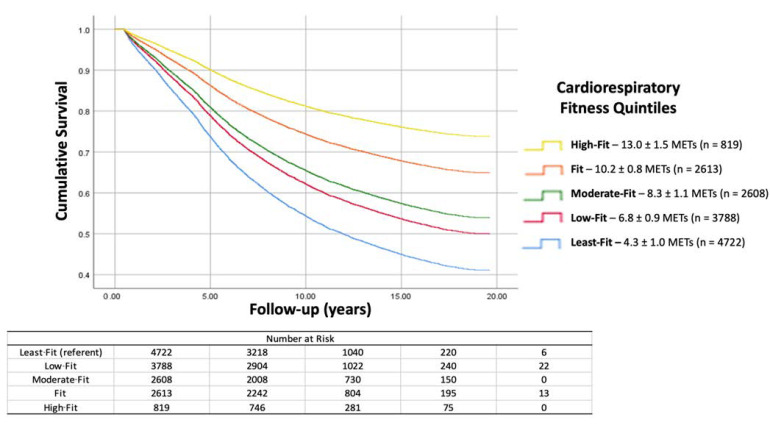
The cumulative hazards for mortality risk across CRF categories are illustrated with survival curves. At the mean follow-up, ten years after surgery, more than 80% of High-Fit patients are alive compared to approximately 55% of patients in the Least-Fit category. CABG = Coronary Artery Bypass Grafting.

The association between CRF and mortality after CABG was inverse and graded. There was improved survival with increased CRF across the spectrum of fitness levels. Each 1-MET increase in CRF was associated with an 11% lower mortality risk. Hazard ratios for mortality for each of the five fitness categories are presented in Table 3. Hazard ratios were similar for patients who had a prior diagnosis of cardiovascular disease in comparison to those who did not. Time from CABG to death varied significantly between CRF quintiles: Least-Fit = 7.1 ± 4.2 years, Low-Fit = 7.8 ± 4.1 years, Moderate-Fit = 8.0 ± 4.2 years, Fit = 8.6 ± 3.9 years, High-Fit = 9.1 ± 3.9 years. The cumulative hazards for mortality risk across CRF categories are illustrated with survival curves in Figure 2.

## 4. Discussion

Cardiorespiratory fitness has long been established as a significant factor influencing all-cause mortality, the development of CAD, and the incidence of CVD events such as MI. The major new finding in the present study is the graded inverse relationship between fitness and long-term mortality after CABG. This is the first study to assess the relationship between directly measured CRF and long-term survival after CABG and to establish CRF as a risk factor for long-term mortality after CABG.

These data demonstrated increased survival with higher fitness across the spectrum of fitness levels. The long-term mortality difference between the Least-Fit and Low-Fit groups, for instance, was similar to the long-term mortality difference between the Fit and High-Fit groups. Every 1-MET increase in exercise capacity decreased the mortality risk by 11% (HR = 0.89; CI: 0.88–0.90; *p* < 0.001). This finding suggests that even slight improvements in CRF, regardless of baseline fitness level, could potentially lead to improved long-term outcomes after CABG. There is a significant survival advantage for a relatively modest CRF level that most adults can likely attain by adhering to national physical activity guidelines [25,26].

It has been debated in the literature whether it is the amount of physical activity or the resultant increased aerobic power capacity that is protective against the development of cardiovascular disease [27]. Moderate to high levels of physical activity and physical fitness both produce long-term health benefits [28]. To date, national guidelines on the role of exercise in the prevention of CVD limit their recommendations to physical activity duration and intensity, with little mention of specific performance goals or parameters [25,26]. Our study did not include any data points on patients’ physical activity patterns, a notable limitation. However, our findings demonstrated a durable survival advantage for a high level of fitness at a single point in time years prior to the need for surgical revascularization.

It has been noted in some studies and suggested by some authors that high amounts of strenuous physical activity and the resultant high level of CRF can be detrimental to cardiovascular health [29,30]. In the present study, the highest quintile of CRF was associated with the lowest long-term mortality after surgery. Our data, therefore, do not support an upper limit to the benefit for increased cardiorespiratory fitness.

Recently, Smenes and the other investigators in the HUNT study demonstrated an inverse and graded relationship between physical activity and survival after CABG similar to our own findings, noting a mortality improvement of 15% for each 1-MET increase in estimated CRF [19]. An important distinction between this study and our own is the manner in which CRF was determined. In the present study, CRF was objectively measured via exercise tolerance testing, whereas Smenes et al. estimated CRF based primarily upon the number of hours patients reported performing light and high intensity physical exercise on a weekly basis. It may, therefore, be appropriate to understand the HUNT study as an analysis of physical activity and the current study as an analysis of CRF. As noted, both studies demonstrated improved survival after CABG.

Smith et al. recently demonstrated improved short-term survival after CABG in a high-fit group in comparison to a low-fit group using outcome data from the STS database [18]. Similar to our own study, these investigators used a measured CRF level attained via maximum capacity exercise tolerance testing on a treadmill. Unlike our own study, follow-up was limited to perioperative and 30-day outcomes, and the sample size only allowed for stratification into two groups. Taken together, these studies suggest that CRF is independently associated with improved short- and long-term survival after CABG.

It is important to note that while participants whose ETT was limited by angina or who had EKG evidence of ischemia during ETT were excluded from our study, 55% of the patients in our cohort (8019/14,550) had an established diagnosis of CVD, and 5.9% (862/14,550) had undergone a previous PCI. It is well established that aerobic exercise is beneficial after a diagnosis of CAD and after a CAD event such as MI, and participation in structured cardiac rehabilitation is strongly supported by national guidelines [31,32,33,34]. Our data provide additional support for these guidelines, particularly for patients with existing CAD and a prior PCI, who may derive a survival advantage with greater fitness levels should they go on to require CABG.

The strong relationship between CRF and survival suggests a potential mortality benefit from an exercise program aimed at increasing CRF. Such a program would best be prescribed by an exercise physiologist and supervised and encouraged by a primary care physician or preventative cardiologist. It is not currently the authors’ practice to prescribe exercise regimens or to make routine referrals to an exercise physiologist for patients referred for CABG.

Age is an important covariate to consider in any study investigating mortality as an end point. In the present study, Least-Fit individuals were approximately 2 years older than the younger group (Fit); however, they were approximately 1 year younger than the oldest group in our cohort (Moderate-Fit), who had a 31% lower risk of mortality in comparison to the Least-Fit group. The models were adjusted for age in addition to other covariates. Thus, it is unlikely that 1 to 2 years of age difference across the CRF groups were responsible for the graded decline in risk.

The amount of time from ETT to CABG differed slightly among the CRF quintiles, with the lowest time recorded in the Least-Fit group and progressively greater time to CABG recorded in each successive fitness quintile. The reason for this is not entirely clear, but we speculate that this is related to the slower progression of coronary artery disease that has been documented in other studies [11,13,14,15].

## 5. Strengths and Limitations

This study has several notable limitations. First, the conclusions of this study may not be applicable to the general population because the study population is limited to primarily male Veterans. It has been demonstrated that the progression of CAD and the factors influencing outcomes after CABG differ between men and women [4,35]. Second, the association between fitness and survival, while compelling, does not indicate causality. Third, the prevalence of comorbid conditions known to impact mortality after CABG was significantly different amongst CRF quintiles. These differences were addressed with statistical methods in the multivariable analysis. However, it remains that chronic illnesses known to be associated with worse survival after CABG were significantly higher in the Least-Fit group and progressively lower in more fit groups. Fourth, because this study includes only a single exercise tolerance test for each subject, we cannot conclude that an intervention to increase CRF, such as an exercise program, would necessarily lead to changes in survival after CABG. Fifth, we have no data on the physical activity of our subjects. Neither do we have data on the development of chronic medical conditions after exercise testing. Lastly, the cause of death of our subjects is not known, so it is not possible to delineate cardiac versus noncardiac deaths.

Exclusion criteria, such as participants completing ETT within six months of an MI, PCI, CABG, or new heart failure diagnosis, strengthened the study by ensuring that participants were not limited by their cardiac disease in completing the ETT. However, the authors recognize that six months is an arbitrary cutoff and may have excluded otherwise appropriate participants.

This study has several notable strengths. With more than 14,000 patients included in the study, the sample size is quite large in comparison to other studies of fitness and outcomes after CABG, allowing for stratification into distinct fitness quintiles. CRF was objectively measured using validated exercise tolerance tests rather than estimated. Follow-up of study participants after surgery averaged ten years, providing excellent long-term mortality data.

## 6. Conclusions

CRF is inversely and independently associated with long-term mortality after CABG in Veterans referred for exercise testing. This association is present at all levels of fitness. CRF is a significant prognostic factor for long-term mortality after CABG. A high level of fitness at a single point in time, even when measured months or years prior to CABG, is associated with improved long-term survival after CABG. Interventions aimed at improving CRF may improve long-term survival after CABG, but more research is necessary in this area. Cardiothoracic surgeons should add their voice to the national call for increased physical activity and CRF for the public.

## Figures and Tables

**Table 1:** Clinical and Demographic Patient Characteristics at the Time of Exercise Tolerance Test by Fitness Group.

Patient Characteristic	Least-Fit (n = 4722)	Low-Fit (n = 3788)	Moderate-Fit (n = 2608)	Fit (n = 2613)	High-Fit (n = 819)	Significance (*p*-Value)
Age at ETT (years)	64.2 ± 8.2	63.3 ± 8.2	65.0 ± 9.0	62.0 ± 7.2	62.5 ± 8.5	<0.001
BMI (kg/m^2^)	30.6 ± 5.7	30.2 ± 5.1	29.4 ± 4.5	29.0 ± 4.3	27.9 ± 4.0	<0.001
Body Weight (kg)	95.7 ± 19.3	94.5 ± 17.9	91.7 ± 15.9	90.4 ± 15.2	86.4 ± 14.0	<0.001
Atrial Fibrillation or Flutter	464 (9.8%)	248 (6.5%)	179 (6.9%)	123 (4.7%)	29 (3.5%)	<0.001
Chronic Kidney Disease	691 (14.6%)	368 (9.7%)	222 (8.5%)	164 (6.3%)	29 (3.5%)	<0.001
Diabetes Mellitus	1810 (38.3%)	1158 (30.6%)	663 (25.4%)	477 (18.3%)	100 (12.2%)	<0.001
Cardiac/Hypertension Medications	4171 (88.3%)	3181 (84.0%)	2161 (82.9%)	1968 (75.3%)	562 (68.6%)	<0.001
CVD including CAD	2878 (60.9%)	2117 (55.9%)	1401 (53.7%)	1267 (48.5%)	349 (42.6%)	<0.001
Smoking	1386 (29.4%)	959 (25.3%)	516 (19.8%)	629 (24.1%)	176 (21.5%)	<0.001
History of Stroke	69 (1.5%)	28 (0.7%)	28 (1.1%)	10 (0.4%)	5 (0.6%)	<0.001
Hypertension	3960 (83.9%)	2989 (78.9%)	2032 (77.9%)	1866 (71.4%)	514 (62.8%)	<0.001
Statin Use	2948 (36.6%)	2125 (56.1%)	1360 (52.1%)	1253 (48.0%)	361 (44.1%%)	<0.001

ETT = Exercise Tolerance Test; BMI = Body Mass Index; CVD = Cardiovascular Disease; CAD = Coronary Artery Disease.

**Table 2:** Notable Clinical and Demographic Variables and Their Association with Long-Term Mortality after CABG.

Patient Characteristic	Hazard Ratio	95% Confidence Interval	*p*-Value
Atrial Fibrillation or Flutter	1.60	1.52–1.69	<0.001
Chronic Kidney Disease	1.53	1.42–1.64	<0.001
Diabetes Mellitus	1.44	1.37–1.52	<0.001
Cardiac/Hypertension Medications	1.27	1.16–1.39	<0.001
CVD including CAD	1.21	1.15–1.27	<0.001
Smoking	1.14	1.08–1.21	<0.001
History of Stroke	1.14	0.92–1.42	0.243
Age	1.04	1.04–1.05	<0.001
Hypertension	1.03	0.95–1.10	0.519
BMI	0.97	0.97–0.98	<0.001
Statin Use	0.93	0.89–0.99	0.012
History of PCI	0.83	0.75–0.93	0.001

CABG = Coronary Artery Bypass Graft; CVD = Cardiovascular Disease; CAD = Coronary Artery Disease; BMI = Body Mass Index.

**Table 3:** Mortality Risk According to Cardiorespiratory Fitness Quintile.

Cardiorespiratory Fitness Quintile	Hazard Ratio	95% Confidence Interval	*p*-Value
Entire Cohort (n = 14,550)			
Least-Fit (referent)	1.00	--	--
Low-Fit	0.76	0.72–0.81	<0.001
Moderate-Fit	0.66	0.62–0.71	<0.001
Fit	0.47	0.44–0.51	<0.001
High-Fit	0.33	0.29–0.38	<0.001
No Known CVD (n = 6538)			
Least-Fit (referent)	1.00	--	--
Low-Fit	0.76	0.69–0.83	<0.001
Moderate-Fit	0.63	0.56–0.7	<0.001
Fit	0.45	0.40–0.51	<0.001
High-Fit	0.33	0.27–0.41	<0.001
Known CVD (n = 8012)			
Least-Fit (referent)	1.00	--	--
Low-Fit	0.77	0.71–0.82	<0.001
Moderate-Fit	0.69	0.63–0.75	<0.001
Fit	0.49	0.45–0.54	<0.001
High-Fit	0.34	0.28–0.41	<0.001

CVD = Cardiovascular Disease.

## Data Availability

Please contact the corresponding author for requests regarding data availability.
